# Customized Multiwavelets for Planetary Gearbox Fault Detection Based on Vibration Sensor Signals

**DOI:** 10.3390/s130101183

**Published:** 2013-01-18

**Authors:** Hailiang Sun, Yanyang Zi, Zhengjia He, Jing Yuan, Xiaodong Wang, Lue Chen

**Affiliations:** 1 State Key Laboratory for Manufacturing and Systems Engineering, Xi'an Jiaotong University, Xi'an 710049, Shaanxi, China; E-Mails: hailiang41@live.cn (H.S.); hzj@mail.xjtu.edu.cn (Z.H.); yuanjing_802@163.com (J.Y.); xjtuwxd@gmail.com (X.W.); 2 Shanghai Institute of Radio Equipment, Shanghai 200090, China; 3 Technology Center, CNPC Logging Co., Xi'an 710077, Shaanxi, China; 4 Beijing Aerospace Control Center, Beijing 100094, China; E-Mail: luechen0912@yahoo.com.cn

**Keywords:** planetary gearbox, fault detection, vibration sensor signals, customized multiwavelets, redundant symmetric lifting schemes, improved neighboring coefficients

## Abstract

Planetary gearboxes exhibit complicated dynamic responses which are more difficult to detect in vibration signals than fixed-axis gear trains because of the special gear transmission structures. Diverse advanced methods have been developed for this challenging task to reduce or avoid unscheduled breakdown and catastrophic accidents. It is feasible to make fault features distinct by using multiwavelet denoising which depends on the feature separation and the threshold denoising. However, standard and fixed multiwavelets are not suitable for accurate fault feature detections because they are usually independent of the measured signals. To overcome this drawback, a method to construct customized multiwavelets based on the redundant symmetric lifting scheme is proposed in this paper. A novel indicator which combines kurtosis and entropy is applied to select the optimal multiwavelets, because kurtosis is sensitive to sharp impulses and entropy is effective for periodic impulses. The improved neighboring coefficients method is introduced into multiwavelet denoising. The vibration signals of a planetary gearbox from a satellite communication antenna on a measurement ship are captured under various motor speeds. The results show the proposed method could accurately detect the incipient pitting faults on two neighboring teeth in the planetary gearbox.

## 1. Introduction

**Figure 1.**
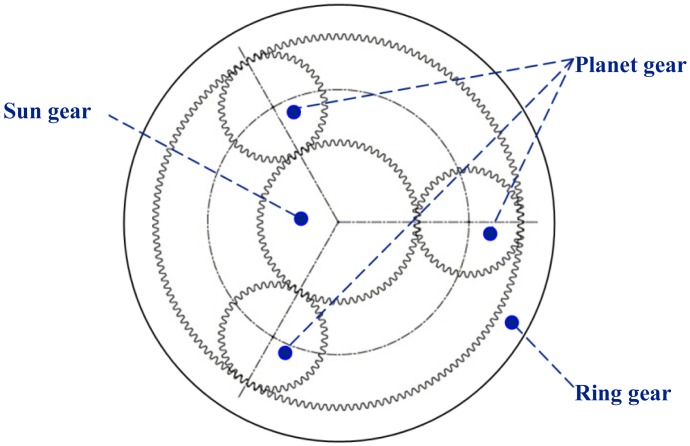
Schematic of an elementary planetary gear set having three planet gears.

Accurate fault detection of planetary gearboxes is important to reduce unscheduled machine downtime and avoid catastrophic accidents [1]. As key components, planetary gearboxes have been widely used in automotive, aerospace and heavy industry applications such as helicopters, wind turbines and mining machines because they have the advantages of large transmission ratios, strong load-bearing capacity and high transmission efficiency [2]. However, planetary gearboxes inevitably generate various faults because of long term running under complex and severe conditions such as heavy load, fatigue, corrosion and elevated temperature. As shown in Figure 1, an elementary planetary gear set [3] is composed of a sun gear, an internal or ring gear and several identical planet gears located around the sun gear. The planet gears are held by a common rigid structure, called planet carrier through planet bearings. In Figure 1, the ring gear is fixed, the sun gear rotates around its own center, the planet gears rotate around their own centers and revolve around the center of the sun gear.

With a special gear transmission structure, planetary gearboxes exhibit complicated dynamic responses which are more difficult to detect than fixed-axis gear trains [4]. It is because multiple planet gears produce similar vibrations and these similar vibrations with different meshing phases couple with each other [5,6]. Researchers have found that compound vibration transmission paths from the gear mesh points to the acceleration sensors may deteriorate or attenuate vibration responses of gear faults through dissipation, interference and resonance effects [7]. Besides, abundant work indicates that most of the vibration energy occurs at various sidebands of the gear meshing frequency and its harmonics [8] and nonlinear transmission path effects caused by the torques or loads would weaken the fault features hidden in vibration signals [5]. These complicated dynamic responses increase the difficulty of planetary gearbox fault detection and reduce the effectiveness of fault diagnosis methods for fixed-axis gearboxes when applied to planetary gearboxes.

Up to now, researchers have proposed a few interesting methods based on advanced signal processing techniques for detecting planetary gearbox faults. Blunt and Keller [5] developed the planet carrier method and planet separation method to detect a fatigue crack in a planet carrier of an epicyclic transmission, which was a component of the main transmission gears in the US Army's UH-60 A Black Hawk helicopters. Barszcz and Randall [9] applied the spectral kurtosis (SK) technique to detect a tooth crack in the planetary gear of a wind turbine. Bartelmus and Zimroz [10,11] introduced the load susceptibility concept for the condition monitoring of planetary gearboxes under time-variable operating conditions. It was stated that the acceleration signal envelopes showed deeper amplitude modulation for the gearbox in bad condition than that in good condition. Hameed and Hong [12] profoundly reviewed different techniques, methods and algorithms developed to monitor the performances of wind turbines to keep them away from catastrophic conditions caused by sudden breakdowns. Lei and Kong [4] proposed two diagnostic parameters specially designed for fault detection and diagnosis of planetary gearboxes. The two parameters are the root mean square of the filtered signal (FRMS) and the normalized summation of positive amplitudes of the difference spectrum between the unknown signal and the healthy signal (NSDS). Lei and Lin [13] introduced a method based on multisensor information fusion to classify the pitting damages with different levels in a planetary gearbox.

In summary, researches on planetary gearbox fault diagnosis have only focused on the condition monitoring and fault classifications. Studies on weak feature detections of incipient faults are rare and these weak features are always immersed in noises generated by the equipment and the surrounding environment. It is significant to detect weak fault features as early as possible, which is a complicated and challenging task that requests advanced analytical methods with high reliability, high accuracy and high efficiency.

The emerging notion of multiwavelet transform (MWT), which uses vector-valued scaling and wavelet functions, is an important development of the wavelet theory. Multiwavelets possess excellent properties of orthogonality, symmetry, compact support and high vanishing moments simultaneously [14,15]. Since 1994, Geronimo-Hardin-Massopust (GHM) multiwavelet [16,17], Chui-Lian (CL) multiwavelet [18] and Hermite multiwavelet [19] have been proposed successively and received considerable attention from wavelet research communities both in theory and in applications. Khadem and Rezaee [20] applied GHM multiwavelet to detect the gearing system faults. Yuan and He [21] proposed multiwavelet sliding window denoising to detect the gearbox fault features of the hot strip finishing mills. Although these methods showed their advantages over scalar wavelets, prior researches always selected mother multiwavelets from a library of previously designed multiwavelets. However, the chosen standard and fixed multiwavelets were usually not the suitable ones for specified applications [22].

To overcome the limitations of standard or fixed MWTs, integrating multiwavelets with lifting schemes (LS) is an exciting motivation to construct customized multiwavelets with desired properties. LS, introduced by Sweldens [23,24], is a powerful tool to construct biorthogonal wavelets. It provides a great deal of flexibility and freedom to construct adaptive wavelets by the design of prediction operators and update operators. Wang and Zi [25] proposed the customized multiwavelets originated from Hermite splines via symmetric lifting schemes. Yuan and He [26] proposed a method incorporating customized multiwavelet with sliding window denoising, which was an effective and promising tool for gear fault detection.

It is a challenging task to detect weak features of incipient faults, which are always immersed in heavy noises generated by the surrounding environment or the equipment. Multiwavelet denoising plays an important role in eliminating noise as much as possible. Its effect mainly depends on the feature separation by using multiwavelets and the threshold denoising. A redundant multiwavelet possesses the time invariant property [27] and provides abundant information for feature detection of periodical impulses. Symmetry is another important property which avoids the phase error in MWT. To ensure the time invariant and symmetry property of multiwavelets, a method integrating the symmetric lifting scheme and redundant multiwavelet is proposed to construct customized multiwavelets. Then a critical problem is how to evaluate the obtained multiwavelets and to select the optimal ones for specific applications. The quotient of kurtosis and entropy is proposed to select the optimal multiwavelets because kurtosis is sensitive to sharp impulses of incipient faults and entropy is effective for periodic impulses of moderate or severe faults. Furthermore, based on the correlation of neighboring coefficients, the improved neighboring coefficients (INC) [28] is adopted to eliminate noises from the decomposed signals.

In this paper, a method which incorporates the customized multiwavelets and INC is proposed for fault detections of planetary gearboxes. The experimental results show that the proposed method is effective and promising to detect these weak impulse features. The rest of the paper is organized as follows: The theory of multiwavelets and the symmetric lifting schemes are briefly introduced in Section 2. In Section 3, the redundant symmetric lifting scheme is proposed to construct customized multiwavelets and the improved neighboring coefficients is introduced into multiwavelets denoising. In Section 4 experimental results are performed. The conclusions are summarized in Section 5.

## 2. The Theory of Multiwavelets and the Symmetric Lifting Schemes

### 2.1. Multiwavelet Transform

Like scalar wavelets, the theory of multiwavelets is based on multiple resolution analysis (MRA) [15]. The difference is that multiwavelets are generated by dilations and translations of the vector functions ***Φ*** = [*φ_1_*,*φ_2_*, …,*φ_r_*]^T^, where *r* ∈ *N* and the space *V_j_* at scale *j* is:

(1) $$V_{j}=\text{clos}\{2^{j/2}\varphi_{i}(2^{j}\;t-k):1\leq i\leq r,k\in Z\}$$

where *i* is the translation of ***Φ***.

The vector functions ***Φ*** (*t*) and ***ψ***(*t*) satisfy the matrix two-scale relation equation:

(2) $$\begin{array}{cc} \Phi (t)=\sqrt{2}\sum_{k=0}^{M}{\text{H}}_{k}\Phi (2t-k) & k\in \text{Z} \end{array}$$

(3) $$\begin{array}{cc} \Psi (t)=\sqrt{2}\sum_{k=0}^{M}{\text{G}}_{k}\Phi (2t-k) & k\in \text{Z} \end{array}$$

where ***Ψ*** = [*ψ*_1_,*ψ*_2_, …, *ψ*_*r*_]^T^ are multiwavelet functions, ***H****_k_* and ***G****_k_* are lowpass and highpass matrix filter banks, respectively, *k* = 0,1,…, *N* is the number of filter banks. In this paper, the case multiplictity *r* = 2 is under study. By the dilations of Equations (2) and (3), the following recursive relationship between coefficients (*c*_1_*,_j_,_k_*, *c*_2_*,_j_,_k_*)^T^ and (*d*_1_*,_j_,_k_*, *d*_2_*,_j_,_k_*)^T^ can be obtained:

(4) $$\begin{array}{cc} \left(\begin{array}{c} c_{1,j-1,k}\\ c_{2,j-1,k} \end{array}\right)=\sqrt{2}\sum_{n=0}^{K}H_{n}\left(\begin{array}{c} c_{1,j,2k+n}\\ c_{2,j,2k+n} \end{array}\right), & j,k\in Z \end{array}$$

(5) $$\begin{array}{cc} \left(\begin{array}{c} d_{1,j-1,k}\\ d_{2,j-1,k} \end{array}\right)=\sqrt{2}\sum_{n=0}^{K}G_{n}\left(\begin{array}{c} c_{1,j,2k+n}\\ c_{2,j,2k+n} \end{array}\right), & j,k\in Z \end{array}$$

Similarly, multiwavelet reconstruction can be obtained by:

(6) $$\left(\begin{array}{c} c_{1,j,n}\\ c_{2,j,n} \end{array}\right)=\sqrt{2}\sum_{n=0}^{K}\left(H_{k}^{*}\left(\begin{array}{c} c_{1,j-1,2k+n}\\ c_{2,j-1,2k+n} \end{array}\right)+G_{k}^{*}\left(\begin{array}{c} d_{1,j-1,2k+n}\\ d_{2,j-1,2k+n} \end{array}\right)\right)$$

where ***H***_k_* and ***G***_k_* are dual matrices of ***H****_k_* and ***G****_k_*, respectively.

**Figure 2.**
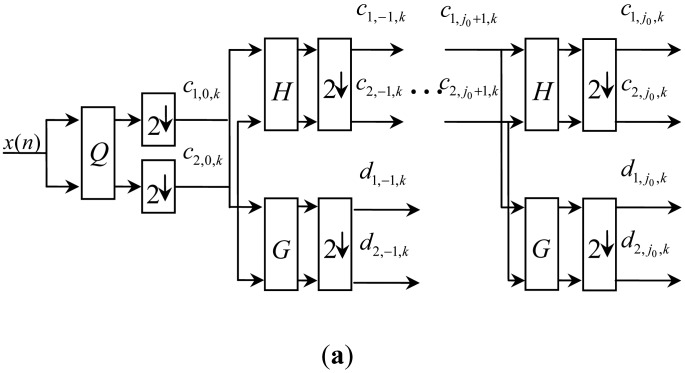

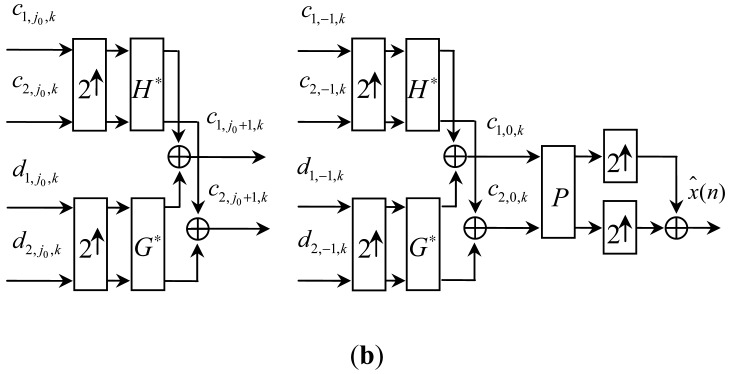
(**a**) Decomposition (*r* = 2) of MWT. (**b**) Reconstruction (*r* = 2) of MWT.

Decomposition and reconstruction of MWT can be represented in Figure 2. In view of the matrix filter banks, preprocessing is necessary for the one stream input signal *x*[*n*]. Correspondingly, a postprocessing method is the inverse process of preprocessing. *Q* represents a prefilter while *P* is a post-filter. {*H*, *G*, H*, G*} represent low-pass filter banks, dual low-pass filter banks, high-pass filter banks, and dual filter banks of multiwavelets. 2 ↓ and 2 ↑ represent decimation and zero-padding, respectively. {*c*_1,0,_*_k_*,*c*_1,−1,_*_k_*,…*c*_1,_*_j_*_0,_*_k_*,*d*_1,_*_j_*_0,_*_k_*} are the first branch and of the decomposition result and {*c*_2,0,_*_k_*,*c*_2,−1,_*_k_*,…*c*_2,_*_j_*_0,_*_k_*,*d*_2,_*_j_*_0,_*_k_*} are the second branch.

Different from scalar wavelets, multiwavelets require two or more input streams because of their matrix filter banks. Usually, there is only one input stream *x*[*n*] and therefore some kind of preprocessing method must be performed before multiwavelet decomposition [29]. Then a postprocessing method is needed to be performed after multiwavelet reconstruction and it is the inverse process of preprocessing. Moreover, different preprocessing methods have significantly different influences on performances of MWT. The most obvious way to get the second input row was just to repeat the input stream with a factor, namely the repeated row as shown in Equation (7). Here *x_k_* is the input signal and *α* is a constant which makes the system hold approximation order higher than zero. Strela [30] claimed that the “repeated row” was effective for feature extraction. Therefore, it is adopted in this paper:

(7) $${\text{s}}_{0,n}=\left(\begin{array}{c} s_{0,n}^{\left(0\right)}\\ s_{0,n}^{\left(1\right)} \end{array}\right)=\left(\begin{array}{c} x_{k}\\ \alpha x_{k} \end{array}\right)$$

Although multiwavelets have several advantages over scalar wavelets, fixed or standard multiwavelets are usually not the optimal for specified applications because they are usually independent of a measured signal [22]. Since different multiwavelets have different time-frequency structures, it is always difficult to choose the optimal ones for specific fault feature extractions. In addition, an unsuitable multiwavelet will reduce the feature detection accuracy. To overcome the limitations, it is necessary to develop new methods to design signal-adapted multiwavelets for mechanical fault diagnosis.

### 2.2. Multiwavelet Lifting Scheme

Lifting scheme [23,24] is a powerful design and implementation technique for conventional wavelets. It provides a great deal of flexibility and freedom for the constructions of biorthogonal wavelets and it is usually used to construct customized wavelets by the design of prediction operators and update operators. Vanishing moment is an important property of multiwavelet functions, which plays a crucial role in the characterization of local Holder exponent of singularities. In theory, a higher vanishing moment means the scaling functions can represent more complex signals accurately. In this paper, lifting scheme was used to improve the existing multiwavelets such that the obtained multiwavelets had enough vanishing moments to describe the singularity of the measured signals.

#### Theorem 2.1

[26] Let the original filter banks of multiwavelets be {***H***(z), ***G***(z), ***H̃***(z), ***G̃***(z)}, new multiwavelet filter banks {***H****_new_*(z), ***G****_new_*(z), ***H̃****_new_*(z), ***G̃****_new_*(z)} are as follows:

(8) $$\begin{array}{l} {\text{H}}_{\text{new}}(z)=\text{H}(z)\\ {\text{G}}_{\text{new}}(z)=\text{T}(z^{2})\left(\text{G}(z)+\text{S}(z^{2})\text{H}(z)\right)\\ {\tilde{\text{H}}}_{\text{new}}(z)=\tilde{\text{H}}(z)-{\text{S}}^{*}(z^{2})\tilde{\text{G}}(z)\\ {\tilde{\text{G}}}_{\text{new}}(z)={\left({\text{T}}^{*}(z^{2})\right)}^{-1}\tilde{\text{G}}(z) \end{array}$$

where the lifting matrices ***S***(*z*) and ***T***(*z*) are finite orders and the determinant of ***T***(*z*) is a monomial. Multiwavelet lifting scheme is similar to scalar lifting scheme except that the filter banks of multiwavelets are matrices, and ***G****_new_*(*z*) contains an extra lifting matrix ***T***(*z^2^*).

According to the two-scale relations and Equation (8), the following formula is obtained:

(9) $$\begin{array}{l} \Phi_{\text{new}}(z)=\Phi (z)\\ \Psi_{\text{new}}(z)=\text{T}(z^{2})\left(\Psi (z)+\text{S}(z^{2})\Phi (z)\right)\\ {\tilde{\Phi }}_{\text{new}}(z)=\tilde{\Phi }(z)-{\text{S}}^{*}(z^{2})\tilde{\Psi }(z)\\ {\tilde{\Psi }}_{\text{new}}(z)={\left({\text{T}}^{*}(z^{2})\right)}^{-1}\tilde{\Psi }(z) \end{array}$$

Lifting scheme can be used to improve the existing multiwavelets. The obtained multiwavelets are considered ideal since they have a desirable vanishing moment. The key of this method is the design of the lifting matrices ***S***(*z*) and ***T***(*z*) [25].

#### Definition 2.2

The *k*-th continuous moments of ***Φ***, ***ψ*** are defined as *M*(***Φ***,*n*) = ∫***Φ***(*x*)*x^n^dx* and *M*(***Ψ***,*n*) = ∫***Ψ***(*x*)*x^n^dx*. Then we have:

(10) $$\begin{array}{c} M(\Phi ,n)=\sum_{j=0}^{n}\sum_{k\in Z}{\text{H}}_{k}\left(\begin{array}{c} n\\ j \end{array}\right)k^{j}M(\Phi ,n-j)\\ M(\Psi ,n)=\sum_{j=0}^{n}\sum_{k\in Z}{\text{G}}_{k}\left(\begin{array}{c} n\\ j \end{array}\right)k^{j}M(\Phi ,n-j) \end{array}$$

Hence, the construction of a wavelet with specified vanishing moments is now a straightforward procedure. An initial wavelet *ω*_0_(*x*) (*ω*_0_(*x*) = *ψ*_1_ or *ψ*_2_) is chosen and a set of *k* translates of scaling functions and wavelet functions *ω*_1_(*x*),…*ω_k_*(*x*) that will be used to modify the function *ω*_0_(*x*) via a lifting step are selected. Then a “lifting coefficient equation” is obtained:

(11) $$\omega_{0}^{\text{new}}=\omega_{0}(x)+\sum_{i=1}^{k}c_{i}\omega_{i}(x)$$

One of the most attractive advantages in multiwavelet lifting is that the functions using to construct new multiwavelets are more than scalar wavelets. When a scalar wavelet is lifted, *ω_i_* is only *Φ*. In the multiwavelet lifting scheme, for *ψ*_1_, *ω_i_* = {*Ψ*_2_,*ϕ*_1_,*ϕ*_2_} and for *ψ*_2_, *ω_i_* = {*ϕ*_1_,*ϕ*_2_}. Obviously, there are more kinds of “brick” in constructing new wavelets. In other words, there are more degrees of freedom in multiwavelet lifting scheme.

Supposing that the vanishing moment *p* of a multiwavelet needs to be lifted to *p*′, both sides of the Equation (11) are performed integral and then a set of linear equations in the matrix form are shown as below:

(12) $$\left[\begin{array}{llll} \int \omega_{1}x^{p}dx & \int \omega_{2}x^{p}dx & \cdots & \int \omega_{k}x^{p}dx\\ \int \omega_{1}x^{p+1}dx & \int \omega_{2}x^{p+1}dx & \cdots & \int \omega_{k}x^{p+1}dx\\ \vdots & \vdots & \ddots & \vdots \\ \int \omega_{1}x^{p^{\prime }-1}dx & \int \omega_{2}x^{p^{\prime }-1}dx & \cdots & \int \omega_{k}x^{p^{\prime }-1}dx \end{array}\right]\;\left[\begin{array}{c} c_{1}\\ c_{2}\\ \vdots \\ c_{k} \end{array}\right]=\left[\begin{array}{l} -\int \omega_{0}x^{p}dx\\ -\int \omega_{0}x^{p+1}dx\\ \;\vdots \\ -\int \omega_{0}x^{p^{\prime }-1}dx \end{array}\right]$$

The integrals can be calculated through Equation (10). The solutions {*c_i_*} of this matrix are the coefficients of functions to implement the lifting. The Equation (11) is performed z-transform and the lifting scheme is obtained successfully.

### 2.3. The Symmetric Lifting Scheme

Symmetry is an important property for multiwavelets. It could ensure the filter banks possess linear phase or generalized linear phase, which avoids the reconstruction error. However, the algorithm described above could not ensure the symmetry of multiwavelet lifting scheme. To guarantee the symmetry, a “symmetric selection” method is adopted to select the translation k of multiwavelet functions. Suppose the multiscaling functions *ϕ*_1_,*ϕ*_2_ and multiwavelets *ψ*_1_,*ψ*_2_ are symmetric or anti-symmetric about the points *a_ϕ,_*_1_,*a_ϕ_*_2_,*a_Ψ,_*_1_,*a_Ψ_*_2_ respectively. Thus, the “symmetric selection” is shown as below [25].

Take *ψ*_1_ for example:

(13) $$\begin{array}{l} a_{\Psi_{1}}-(a_{\phi_{i}}+k_{\phi_{i},j,1})=(a_{\phi_{i}}+k_{\phi_{i},j,2})-a_{\Psi_{1}}\\ a_{\Psi_{1}}-(a_{\Psi_{2}}+k_{\Psi_{2},j,1})=(a_{\Psi_{2}}+k_{\Psi_{2},j,2})-a_{\Psi_{1}} \end{array}$$

where *i* = 1,2, j = 1,2, ,*k* ∈ *Z*.

Suppose *B_ϕ_*_1_, *B_ϕ_*_2_, *B_Ψ_*_1_, *B_Ψ_*_2_ = ±1 (1 means symmetry and -1 means anti-symmetry) stand for the symmetry of multiple scaling functions and multiwavelets respectively. *M*(***Φ***,*k*,*n*) = ∫***Φ***(*x*+*k*)*x^n^dx* and *M*(***Ψ***,*k*,*n*) = ∫***Ψ***(*x*+*k*)*x^n^dx* are substituted to Equation (12):

$$\begin{array}{c} M=\left[\begin{array}{llllll} M(\phi_{i},k_{\phi_{i},j,1},p) & M(\phi_{i},k_{\phi_{i},j,2},p) & \cdots & M(\psi_{2},k_{\psi_{2},j,1},p) & M(\psi_{2},k_{\psi_{2},j,2},p) & \cdots \\ M(\phi_{i},k_{\phi_{i},j,1},p+1) & M(\phi_{i},k_{\phi_{i},j,2},p+1) & \cdots & M(\psi_{2},k_{\psi_{2},j,1},p+1) & M(\psi_{2},k_{\psi_{2},j,2},p+1) & \cdots \\ \vdots & \vdots & \ddots & \vdots & \vdots & \vdots \\ M(\phi_{i},k_{\phi_{i},j,1},p^{\prime }-1) & M(\phi_{i},k_{\phi_{i},j,2},p^{\prime }-1) & & M(\psi_{2},k_{\psi_{2},j,1},p^{\prime }-1) & M(\psi_{2},k_{\psi_{2},j,2},p^{\prime }-1) & \cdots \end{array}\right]\\ B=\left[\begin{array}{cccc} 1 & & & \\ B_{\psi_{i}}B_{\phi_{i}} & & 0 & \\ & \ddots & & \\ & & 1 & \\ 0 & & B_{\psi_{1}}B_{\psi_{2}} & \\ & & & \ddots \end{array}\right] \end{array}$$

Let *M_B_* = *MB*, *C* = [*c_Ψ_1__*,*_ϕ_i__*_,1_, …,*c_Ψ_1__*,*_Ψ_2__*_,1_,… ]^T^ and *M_Ψ_* = [*M*(*Ψ_i_*,0,*p*), *M*(*Ψ_i_*,0,*p*+1),…*M*(*Ψ_i_*,0,*p′*−1) ]^T^, then:

(14) $$M_{B}C=M_{\Psi }$$

The solutions of Equation (13) are the coefficients that are used to lift *Ψ*_1_. The lifting of *Ψ*_2_ is similar to *Ψ*_1_ except adopting *ϕ*_1_ and *ϕ*_2_ only. The lifting coefficients are substituted into the “lifting coefficients equation”. Then the equation is performed z-transform and the presentation of the lifting scheme is obtained as follows:

(15) $$G_{\text{new}}(z)=T(z^{2})(G(z)+S(z^{2})H(z))$$

New symmetric biorthogonal multiwavelets are constructed with the help of the lifting matrices ***T***(*z*) and ***S***(*z*). Symmetric condition and vanishing moment condition are supplemented to the equation set *M_B_C* = *M_ψ_*. The solution of the equation set is the lifting coefficients. The lifting matrices ***T***(*z*) and ***S***(*z*) are obtained through using z-transform. Finally, substituting the ***T***(*z*) and ***S***(*z*) into the lifting equation, a new multiwavelet is constructed successfully.

### 2.4. Redundant Multiwavelet Transform

Discrete multiwavelet transform (DMWT) is essentially a decimated multiwavelet transform. It is an ideal tool for non-stationary signal processing, while there are still several limitations. First, the decomposition results of DMWT are time-variant due to down sampling. A forward or backward translation of the original signal will generate different decomposition results [31]. Second, the length of the approximation signal is reduced by half after each decomposition. As the decomposition level increases, the information contained in the approximation signal become more scarce and the time resolution is gradually decreasing. Third, wavelet compression or wavelet denoising can exhibit pseudo-Gibbs phenomena [32] in the neighborhood of singularities when signals are reconstructed. To overcome these limitations, a simple but efficient method is redundant multiwavelet transform (RMWT).

**Figure 3.**
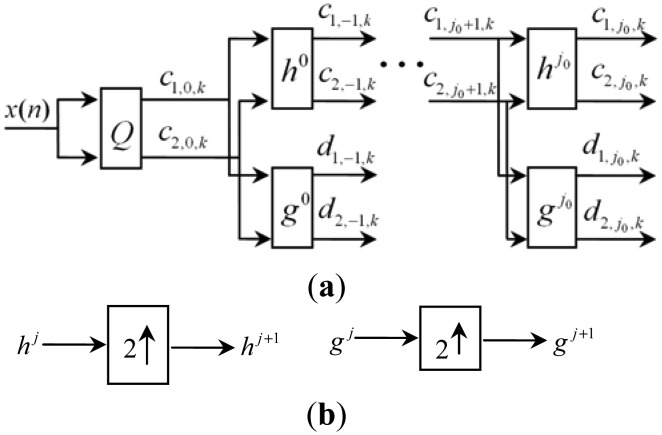
The decomposition of redundant multiwavelet transform. (**a**) Decomposition of redundant multiwavelet transform. (**b**) Zero-padding of filter banks.

RMWT is time-invariant, which is beneficial to the feature extraction of periodic impulses. Moreover, RMWT supply more abundant features and more precise frequency localizations, which are beneficial for mechanical fault detections. The decomposition of RMWT is shown in Figure 3(a). Compared with DMWT, there are no down-samplings in redundant multiwavelet decomposition. The length of the approximation signal and the detail signal after decomposition is the same with the original signals. *h^j^* and *g^j^* are the matrix filters after padding with zeros. Zero-padding of the matrix filter banks is shown in Figure 3(b). *h_i_* and *g_i_* represent the matrix filter coefficients of *h* and *g*, respectively. *i* ∈ *Z* represents the *i - th r* × *r* matrix of two matrix filter banks. The low-pass and high-pass filters of redundant multiwavelet are computed by padding those filters of the original multiwavelets with zeros at the corresponding level l. Suppose *T* is the operator that alternates an arbitrary sequence with zeros, then, for all integers:

(16) $$\begin{array}{cc} (Tx_{2i})=x_{i} & (Tx_{2i+1})= \end{array}0$$

Then the filters of redundant multiwavelet can be calculated by the following equations:

(17) $$\{\begin{array}{l} {\text{H}}_{k}^{l}={\text{G}}_{k}^{l}=0\;\text{if}\;k\;\text{is not a multiple of}\;2^{l},\\ H_{2^{l}i}^{l}={\left(T^{l}\;H\right)}_{2^{l}i}=H_{i}\;G_{2^{l}i}^{l}={\left(T^{l}\;G\right)}_{2_{i}^{l}}=G_{i}\;\text{otherwise}\;. \end{array}$$

## 3. Redundant Symmetric Lifting Schemes and the Improved Neighboring Coefficients

### 3.1. Improved Neighboring Coefficients Denoising

Cai and Silverman [33] proposed a threshold rule incorporating neighbouring coefficients (NC). Chen [34] introduced this method into multiwavelet denoising, which has achieved a wonderful effect in image denoising and mechanical fault diagnosis. The conventional NC procedure chose a constant size of neighboring window *l* = 3 at each level after wavelet decomposition. However, this method is not accurate enough because the size of neighboring window is invariant but the dependences of coefficients are variant at different levels. Wang presented the improved neighboring coefficients denoising after studying the regularity of wavelet coefficients dependency [28], to resolve the problem of constant neighborhood. The formula of incorporating neighboring coefficients is as Equation (18):

(18) $$\begin{array}{cc} S_{j,k}^{2}=\sum_{n=-N}^{N}d_{j,k+n}^{2} & N={\text{N}}_{0}-j \end{array}$$

where *j* is the level of multiwavelet decomposition, the length of neighboring window is 2*N* + 1, N_0_ is a constant, it should be selected according to the signal duration of features and the support of wavelet filters.

**Figure 4.**
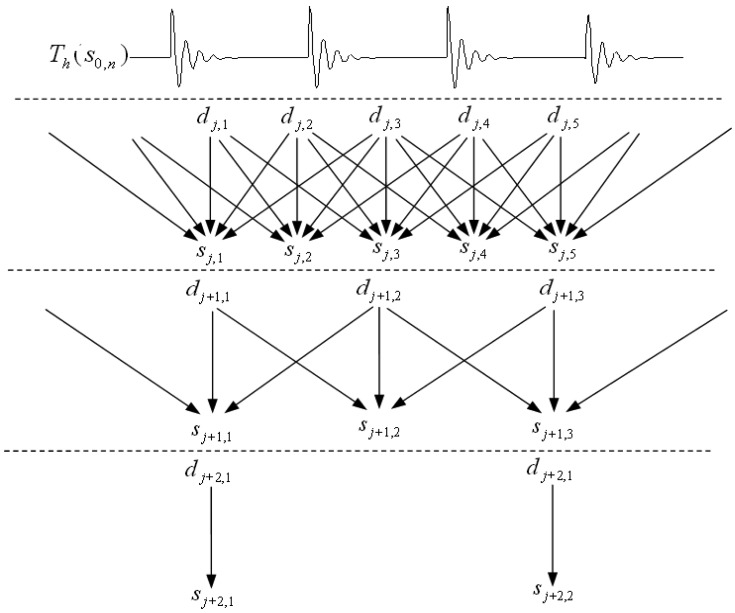
The algorithm of improved neighboring coefficients.

The INC algorithm is shown in Figure 4. It is obvious that the presented method incorporates more coefficients at a low level while less coefficients at a high level. The threshold of INC is shown in Equation (19):

(19) $$d_{j,k}=\{\begin{array}{ll} d_{j,k}(1-\frac{\alpha \lambda_{j}^{2}}{S_{j,k}^{2}}), & S_{j,k}^{2}\geq \alpha \lambda_{j}^{2}\\ 0, & S_{j,k}^{2}<\alpha \lambda_{j}^{2} \end{array}$$

where *λ_j_* = 2log*n_j_*, *α* is an adjusting coefficient of the threshold which is determined by the length of the neighbors, *n* is the length of measured signals. The threshold became term-by-term soft threshold when *N* = 0 while it became conventional neighboring coefficients when *N* = 1.

### 3.2. The Proposed Method Using Customized Multiwavelets and the Improved Neighboring Coefficients

In this paper, redundant symmetric lifting scheme is applied to construct customized multiwavelets with specified properties for specific signals. Then the lifting coefficients of Equation (13) must be underdetermined to ensure there are free parameters in these equations. Generally for a fixed vanishing moment *p*, the more functions are used for the lifting, the longer support of the obtained multiwavelet is. And more functions mean a greater freedom in lifting. However, longer support will reduce the localization of multiwavelet. Therefore, a tradeoff has to be chosen between vanishing moment and numbers of functions used to do lifting.

**Figure 5.**
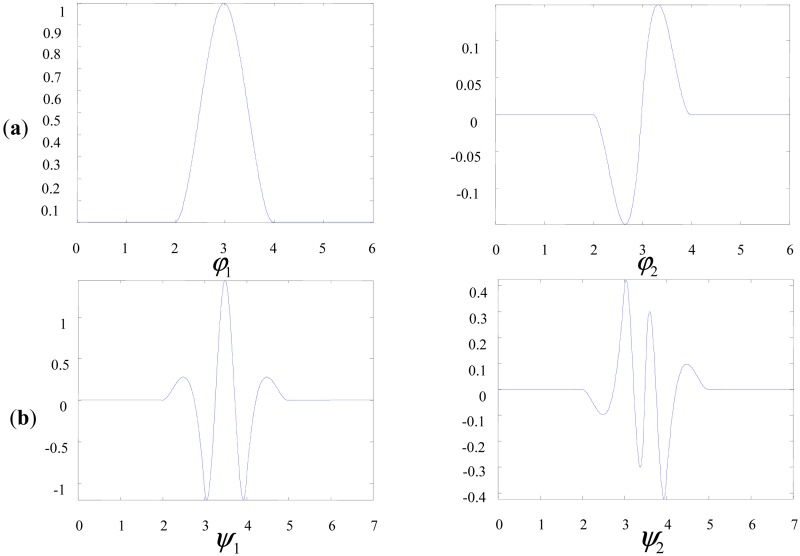
Hermite spline multiscaling functions and multiwavelet functions. (**a**) Multiscaling functions. (**b**) Multiwavelet functions.

We construct new multiwavelet starting from Hermite splines [19] as the original scaling function. In dynamic signals, impulses are always the fault symptoms of defected components and Hermite spline multiwavelet is very similar to impulse components. Multiscaling functions and multiwavelet functions of Hermite spline multiwavelet are shown in Figure 5. There is more freedom and flexibility to construct new multiwavelets with prescribed properties because of the simple waveform of Hermite splines. The proposed methods can act as an effective and promising tool for fault detections of planetary gears.

There are *N_f_* = (*p*′ – *p*) – *Rank*(*M_B_*) free parameters, which are vital for the redundant symmetric lifting scheme. The customized multiwavelet construction is performed by the optimization of the free parameters.

Kurtosis is widely used for fault feature detections because it is sensitive to sharp variant structures, such as impulses. The bigger the impulses in signals, the larger the kurtosis [35]. Furthermore, it is a dimensionless parameter, which is independent of the amplitudes of the signal. The definition of kurtosis is shown in Equation (20):

(20) $$K_{P}=\frac{\sum_{i=1}^{n}{\left(x_{i}-\bar{x}\right)}^{4}}{n\sigma^{4}}$$

where *x_i_* is the *ith* point of the signal *x*, *x̅* is the mean value of *x*, *n* is the signal length of *x*, σ is the standard deviation of *x*. The forth moment of *x* enables the numerator of *K_p_* increases quickly while the denominator increases slowly when incipient faults occurred. Therefore, kurtosis is sensitive for incipient faults with less impulses but descending when impulses are more because of the fault exacerbation. More impulses mean a distinct periodicity. Therefore, kurtosis cannot accurately depict the real signal trend in the detection of periodic shock impulses.

In dynamic response signals, the mechanical fault often expresses as periodic impact features which can be detected through the envelope spectrum of the vibration signal. Hence, the envelope spectrum entropy is selected as an evaluation indicator to obtain the customized multiwavelets.

Assume {*c_i_*}*_i_*_= 1_*_,_*_…,_*_M_* is the class of the normalized coefficients. When it is divided by $\sum_{i=1}^{M}c_{i}$, {*d_i_*}*_i_*_= 1_*_,_*_…,_*_M_* is obtained:

(21) $$d_{j}=c_{j}/\sum_{i=1}^{M}c_{i}$$

The multiwavelet entropy *En_mwt_* is calculated by:

(22) $${\text{En}}_{\text{mwt}}=-\sum_{i=1}^{M}d_{i}\ln d_{i}$$

According to the information theory, the most uncertain probability distribution has the maximum entropy value, and the entropy value reflects the uniformity of the probability distribution. So *En_mwt_* provides the information about the definite degree of the envelope spectrum. The smaller the value of the envelope spectrum entropy *En_mwt_* is, the more distinct periodic impact features will be. Our objective is to find the optimal multiwavelets by finding the minimum value of envelope spectrum entropy *En_mwt_*.

In order to improve the limitation of kurtosis, the proposed method chooses *KE*, the quotient of kurtosis and entropy, as the performance measurement of the lifting scheme and optimized the lifting coefficients with a genetic algorithm to maximize *KE* of the detail coefficients:

(23) $$\text{KE}=\frac{K_{P}}{{\text{En}}_{\text{mwt}}}$$

Genetic algorithms (GAs) are based on the idea of natural selection. The major advantages are their flexibility and robustness as an adaptive global search method. GAs can deal with highly nonlinear problems and non-differentiable functions, as well as functions with multiple local optima. They are parallel implementation in nature. Thus, they are utilized as the tool to optimize free parameters. According to our experimental experience, GA parameters are set as follows: arithmetic crossover and non-uniform mutation operators are adopted, the range of the parameter is chosen to be [–3,3] except 0, the population scale is set to 50, the number of iteration to 30, the probability of crossover to 0.6 and the probability of mutation to 0.05.

**Figure 6.**
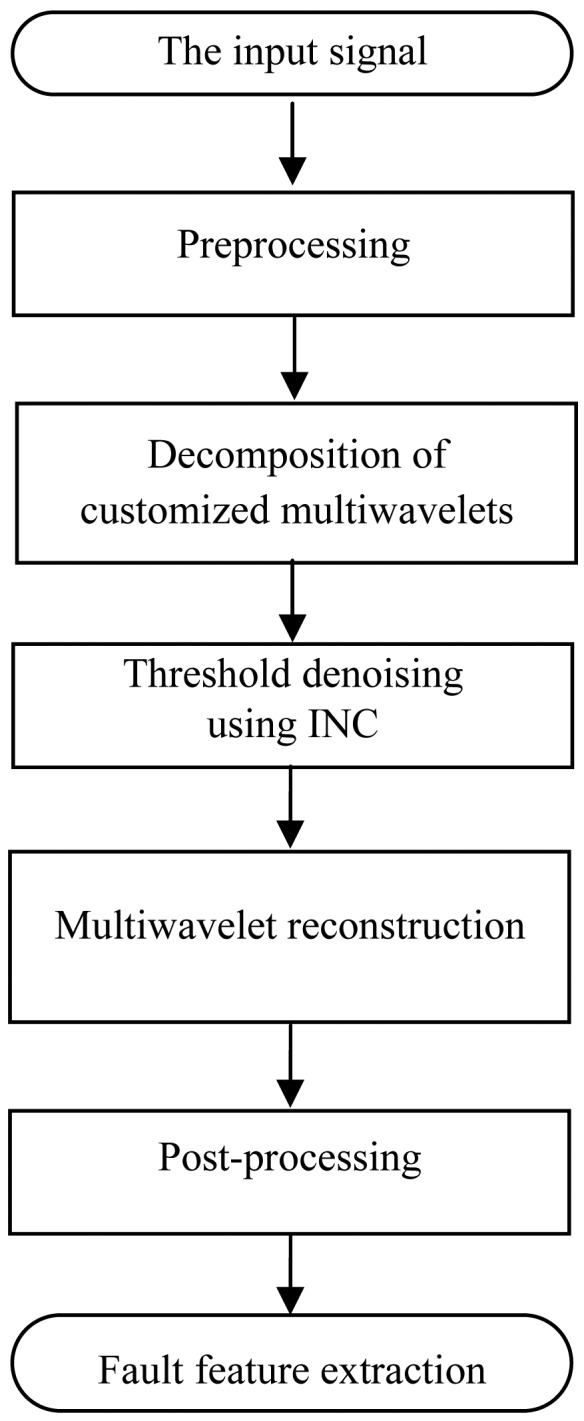
The flow chart of the proposed method.

Figure 6 shows the flow chart of the proposed method using customized multiwavelets and the improved neighboring coefficients (INC). The steps are as follows:

1. Preprocessing method is performed to translate the one-stream input signal into multiple streams.
2. The multiple streams are decomposed by using the customized multiwavelets.
3. Apply INC to shrink the multiwavelet coefficients.
4. The thresholded multiwavelet coefficients are reconstructed.
5. Post-processing method is performed to translate the multiple streams into one-stream. The denoising result is obtained to detect the fault features.

**Figure 7.**
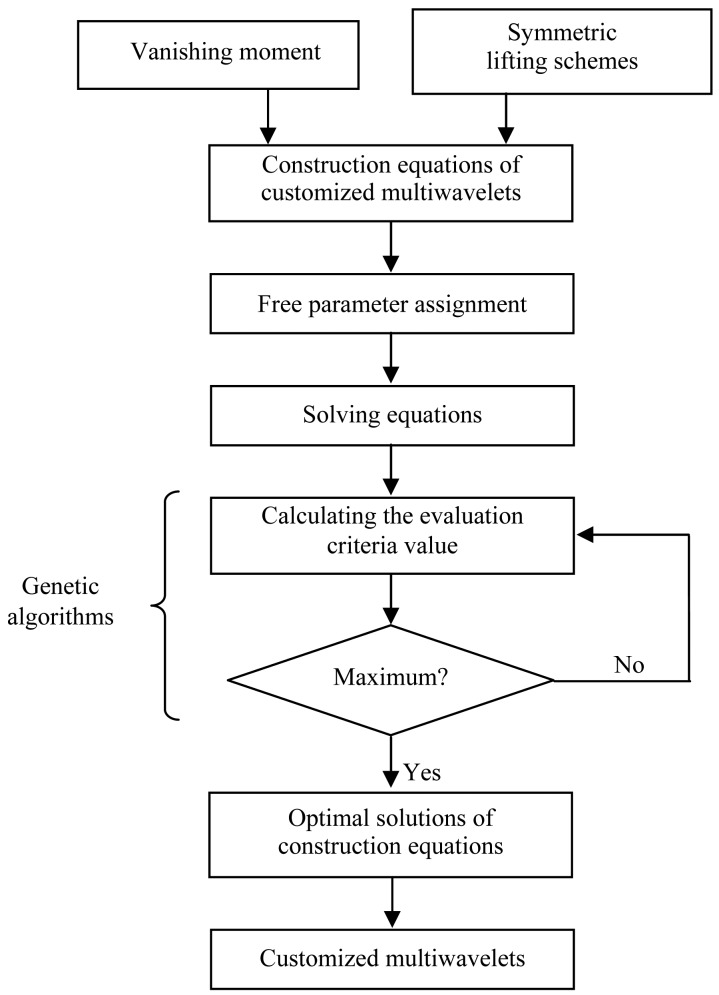
The flow chart of the customized multiwavelets.

Figure 7 shows the flow chart of the customized multiwavelets. First, with the given vanishing moment *p*, the translations of *Φ* and *ψ* are selected using the “symmetric selection” method. Second, assign the values to free parameters and solve the lifting coefficients equations. Third, optimize the free parameters using genetic algorithm. Finally, obtain the optimal symmetric biorthogonal multiwavelet.

In the process of the customized multiwavelet cnostructions, optimization is an important step for modifying the coefficients to match the signal. Suppose the free parameters are {*f*_1_,*f*_2_,…*f_Nf_*}. The process of the optimization using GA is shown as below:

1. Initialize the free parameters {*f*_1_,*f*_2_,…*f_Nf_*}.
2. Substitute {*f*_1_,*f*_2_,…*f_Nf_*} into matrix *M_B_* and make the matrix satisfy *Rank*(*M_B_*) = *p′*−*p*.
3. Compute the lifting coefficients and get *T*(*z*) and *S*(*z*).
4. Decompose the signal with new multiwavelet.
5. Compute *KE* of the detail coefficients and compare it with the maximum value.
6. Generate the more optimal values of the free parameters and return to step 2), otherwise finish the computation.

## 4. Experimental Results

### 4.1. Experimental Setup and Data Acquisition

Planetary gearboxes play an important role in the transmission train of a satellite communication antenna or a telemetry, tracking, and command (TT&C) antenna for the aerospace industry. The aerospace measurement ship is mainly responsible for the maritime measurement and control, communication, salvage and recovery of spacecrafts. Satellite communication antennas (SCA) are critical devices of a measurement ship to support voice, data, fax and video integration services. An SCA comprises three axes, which are the azimuth axis, the pitching axis and the crossing axis. Searching satellite is that makes an SCA circumrotate the azimuth axis, the pitching axis and the crossing axis with controllers to change these axis angles, so the antenna can point itself to different satellite in the light of demands. If the SCA direction departs from a satellite or the satellite makes an excursion, it can adjust the SCA to track the satellite signal automatically.

**Figure 8.**
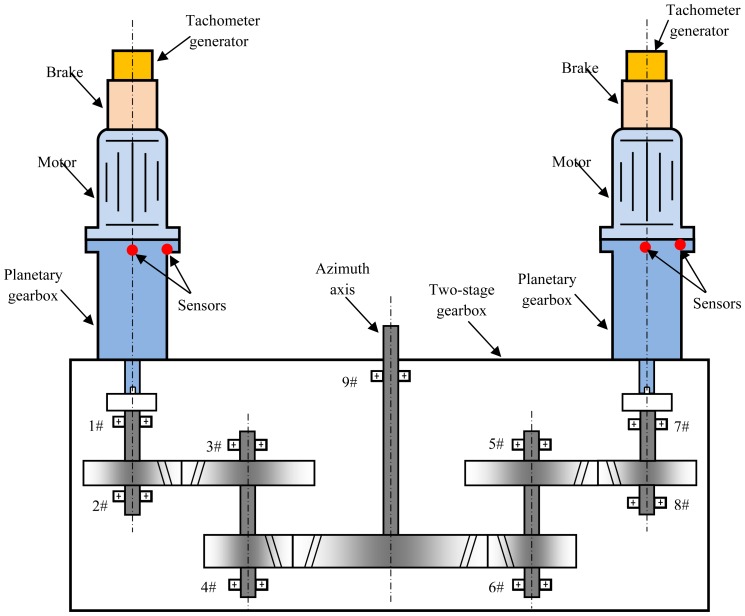
The transmission mechanism of the azimuth axis.

Figure 8 shows the transmission mechanism of the azimuth axis in an SCA. The azimuth axis is fixed, a two-stage gearbox rotates around the azimuth axis. A double-motor dispelling clearance transmission train is designed to raise the transmission accuracy. Each transmission train consists of a tachometer generator, a brake, a motor, a planetary gearbox and a two-stage fixed-shaft gearbox. Planetary gearboxes inevitably generate various faults because of chronically running under such complex and severe conditions as heavy load, ocean wave and tide, fatigue and corrosion. As the core components in the transmission train of an SCA, the performance of planetary gearboxes directly influences the success or failure of these tasks for an SCA.

**Figure 9.**
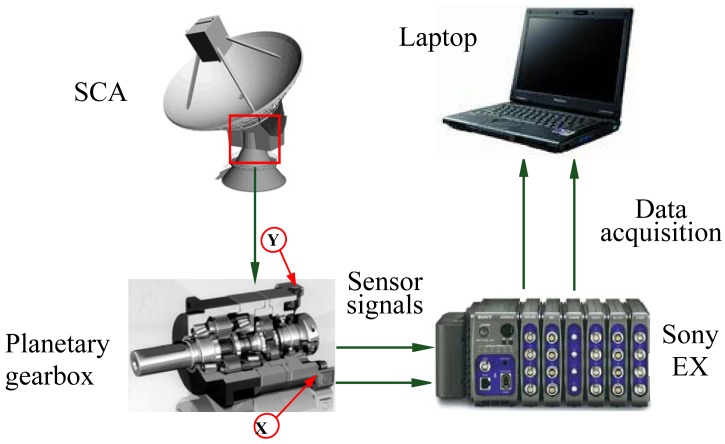
The testing framework of the SCA of a measurement ship.

The testing framework is shown in Figure 9 when a measurement ship was sailing at sea. The left transmission train of the azimuth axis had a slightly abnormal sound when the measurement ship was sailing. The operators replace the defective planetary gearbox with a normal one. Vibration signals are measured before and after the replacement of the planetary gearboxes. Equipment operators controlled the SCA to search and track satellite by using the manual mode. We collected the vibration signals of the transmission trains of the azimuth axis, the pitching axis and the crossing axis using internal electronics piezoelectric (ICP) acceleration sensors. The parameters of these sensors are shown in Table 1. X and Y represented the horizontal and vertical measurement points of the planetary gearbox in the left transmission train of the azimuth axis, respectively. The vibration signals were measured using Sony EX data acquisition system when the SCA was running. A four-order elliptic filter with an anti-aliasing filtering up to 5,500 Hz was adopted. The drive motor was a permanent magnet synchronous AC motor, which was running at two different motor speeds, 150 r/m and 255 r/m. The motor speeds were measured by the tachometer generator at the end of the AC motors.

The planetary gearbox of the azimuth axis transmission train is a two-stage gearbox. The carrier of the first stage works as the input shaft of the second stage. The parameters of the planetary gearbox are shown in Table 2. The number of planet gears in each stage is three.

With the special gear transmission structure, the transmission ratio is calculated using the primary principle of the conversion mechanism method. The first stage in the planetary gearbox has a transmission ratio 8. The meshing frequency of the first stage is calculated by using Equation (24):

(24) $$f_{\text{mesh}}=(N_{in}-N_{\text{out}})*{\text{Z}}_{\text{sun}}/60$$

where *f_mesh_* represents the meshing frequency of the first stage, *N_in_* represents the input speed of the sun gear, *N_out_* represents the output speed of the carrier, *Z_sun_* is the teeth number of the sun gear. According to these parameters, the meshing frequency of the first stage is about 10.5 times of the input speed of *f_in_*.

### 4.2. The Experimental Results of the Normal Planetary Gearbox

**Figure 10.**
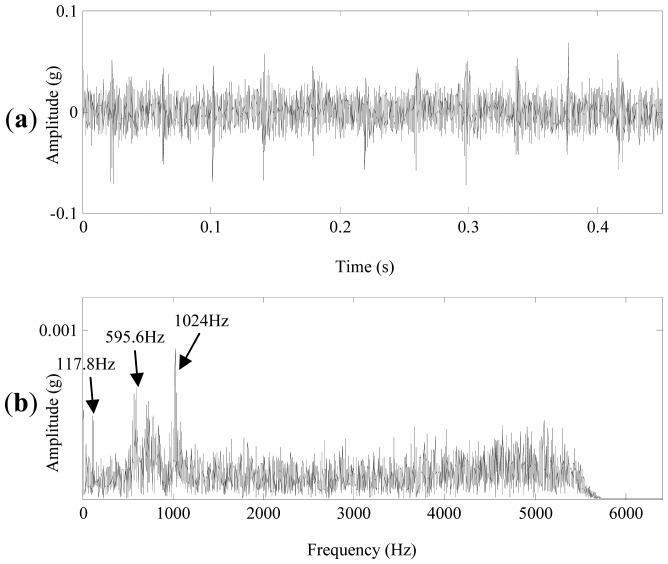
(**a**) The vibration signal of the normal planetary gearbox. (**b**) The FFT spectrum.

Vibration signals of a normal planetary gearbox in the left transmission train of the azimuth axis were acquired. They are applied to verify the effectiveness of the propose method. The sampling frequency and signal length were 12.8 kHz and 5,760, respectively. The rotating speed of the motor was 150 r/m. The signal in time domain is shown in Figure 10(a), the peak to peak value of the signal is about 0.15 g. Its FFT spectrum is plotted in Figure 10(b), the frequency components are abundant. Evident rotating frequencies, meshing frequencies and sidebands relevant to gearbox faults can hardly be identified in the spectrum.

**Figure 11.**
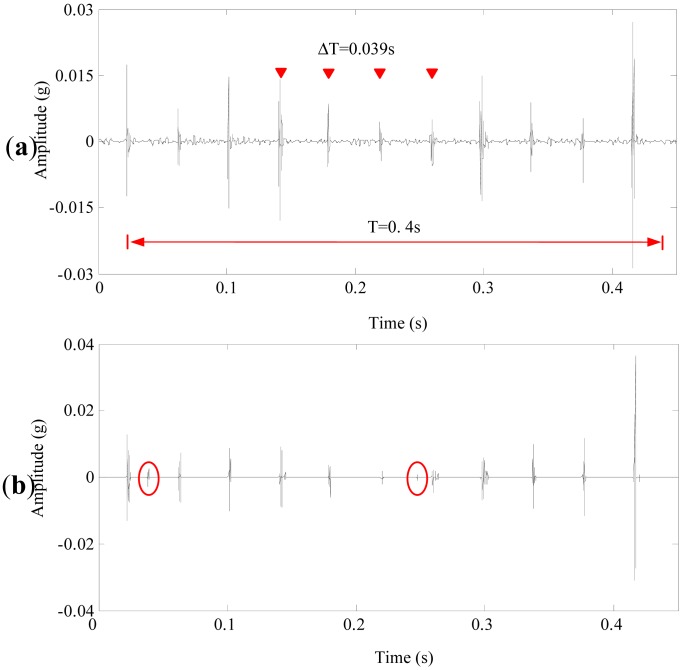
The denoising results of the normal planetary gearbox signal using the customized multiwavelet. (**a**) The proposed method (**b**) Conventional neighboring coefficients.

**Figure 12.**
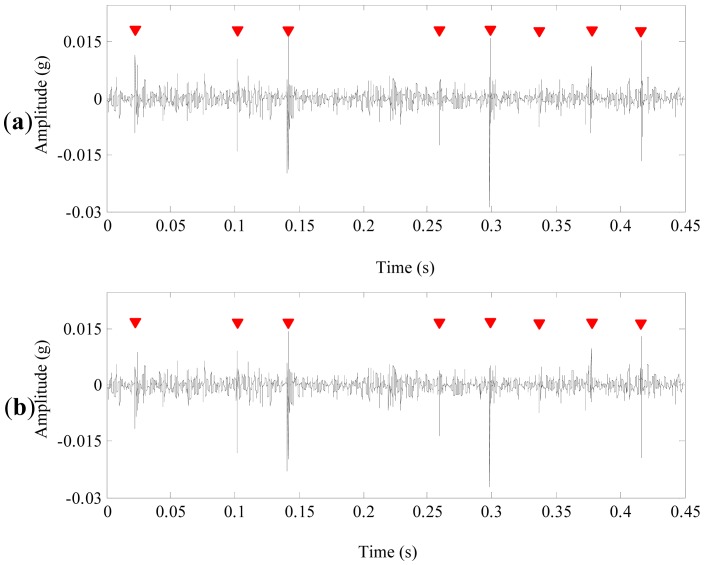
The denoising results of the normal planetary gearbox signal using GHM multiwavelet. (**a**) Improved neighboring coefficients. (**b**) Conventional neighboring coefficients.

The proposed method chose the customized multiwavelet by using redundant symmetric lifting scheme and the optimal threshold by using INC and it was applied to the measured signal. The signal was decomposed into four levels. As shown in Figure 11(a), the proposed method can depict the distinct features with a period of 0.039 s, that is 25.64 Hz. It is about 10.5 times of the rotation speed of the sun gear in the first stage, which is 2.5 Hz. Figure 11(b) displays the denoising result using customized multiwavelet and conventional neighbouring coefficients with an invariant size of neighbors at different decomposition levels. The impulses between 0.16 s and 0.3 s are very weak, and are nearly “over-killed” by the chosen threshold. In addition, unrelated features denoted in the ellipses appear in the denoising result, and these features may influence the validity of fault feature detections. Figure 12(a,b) are the analysis results of GHM multiwavelet denoising using INC and NC, respectively. The denoising results of these two methods are similar, only strong impulses can be detected as shown in the triangles and several singular points appear during 0.02 s and 0.1 s. The impulses in the denoising results are not periodic, which affect the correct fault detections of planetary gearboxes.

According to the kinetic principles of planetary gearboxes, both contact conditions by means of rolling and sliding exist on the gear tooth when meshing. The force of sliding friction changed its direction at the meshing points, which caused the shocking line impacts. Moreover, when each pair of gear tooth got in or out of contact, the load and deformation of each gear increased or decreased suddenly, causing the meshing shocks. The dynamic load consisted of shocking line impacts and meshing shocks, which caused the meshing vibration of gears. Therefore, the meshing vibration in Figure 11(a) is the inherent dynamic characteristics of gear meshing, it is not relevant with gear defects.

**Figure 13.**
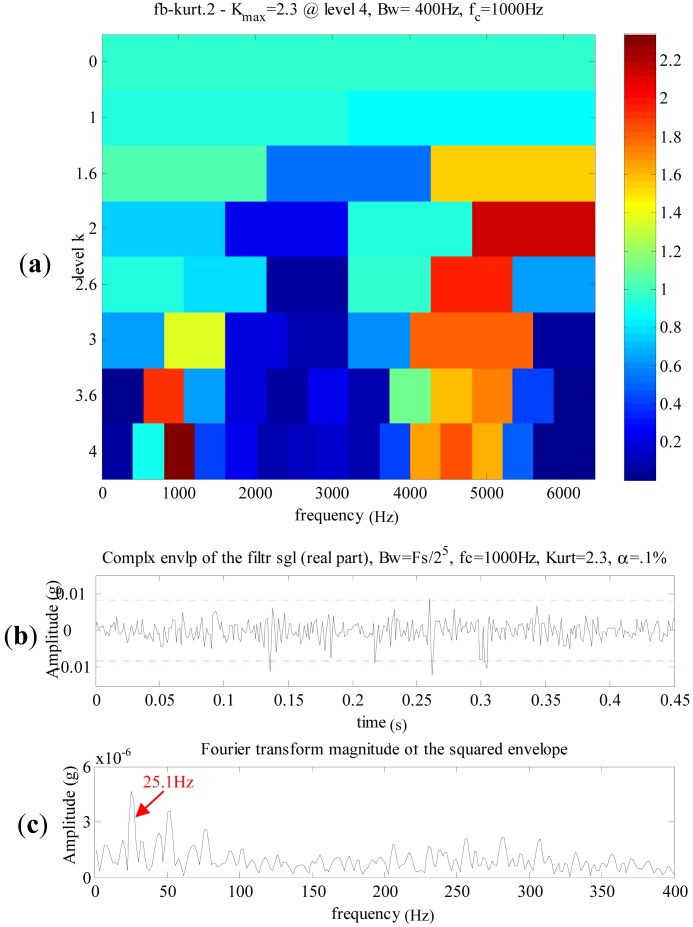
The analyzed results of the normal planetary gearbox signal using spectral kurtosis. (**a**) The Kurtogram. (**b**) The purified signal. (**c**) The envelope spectrum (low-frequency band).

The Kurtogram [36,37] and the resulting signals of spectral kurtosis are illustrated in Figure 13. The maximum kurtosis is 2.3 at level 4, the central frequency is 1,000 Hz and the bandwidth is 400 Hz. The envelope of the filtered signal and its FFT spectrum are shown in Figure 13(b,c), respectively. Figure 13(c) displays a dominant frequency 25.1 Hz, which is close to the meshing frequency of the first stage in the planetary gearbox. However, spectral kurtosis only detects strong impulses between 0.1 s and 0.35 s in the envelope of the filtered signal, while the weak ones are immersed in strong noises.

### 4.3. The Analysis Results of the Defective Planetary Gearbox

**Figure 14.**
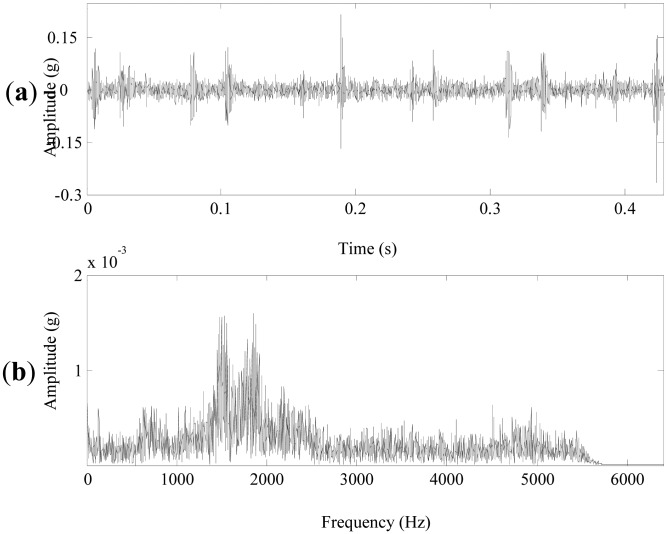
(**a**) The vibration signal of the defective planetary gearbox. (**b**) The FFT spectrum.

The left transmission train of the azimuth axis had a slight abnormal sound when the measurement ship was sailing in the sea. The vibration signals were measured at a sampling frequency of 12.8 kHz from the measuring points on the planetary gearbox by using ICP acceleration sensors. The AC motor was running at 255 r/m. The signal in time domain is shown in Figure 14(a), periodic transient impulses appear in the signal. The peak-to-peak value is about 0.4 g, which is significantly higher than 0.15 g of the normal planetary gearbox. Its FFT spectrum is shown in Figure 14(b), its components mainly distribute at 500∼3,000 Hz. No distinct fault features can be seen in the spectrum.

**Figure 15.**
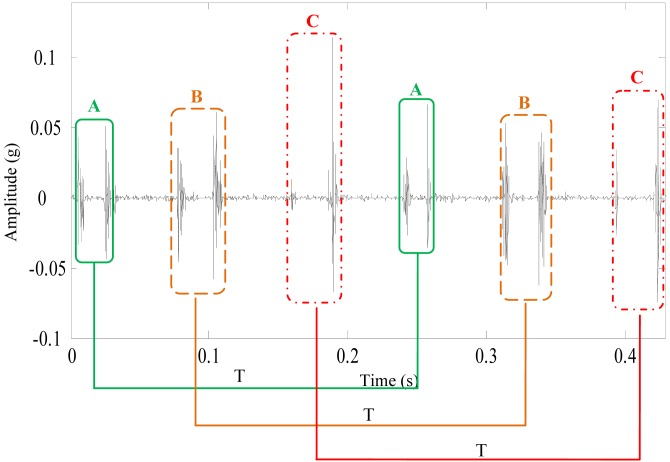
The denoising results of the vibration signal using the proposed method.

The denoising result of the proposed method is shown in Figure 15. During a course of one revolution of the sun gear, three pairs of shock impulses A, B and C occur alternatively. Because there are three planet gears in the first stage of the planetary gearbox. These features indicates that there are several defects in the sun gear. The average time interval between the two neighboring shock impulses in each pair is 0.023 s, corresponding to 43 Hz. It's about 10.5 times of the rotating frequency of the sun gear in the first stage. The frequency 43 Hz is close to the meshing frequency, which suggests that the shock impulses may be caused by defects on two neighboring gear tooth of the sun gear in the first stage.

**Figure 16.**
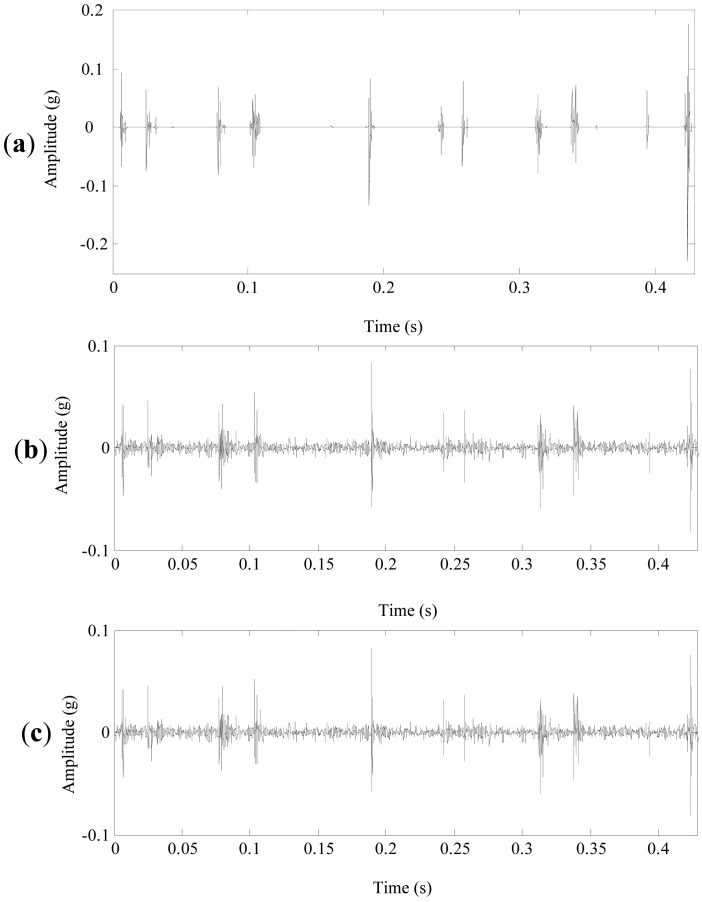
The denoising results of the planetary gearbox signal. (**a**) Customized multiwavelet and NC. (**b**) GHM multiwavelet + INC. (**c**) GHM multiwavelet + NC.

**Figure 17.**
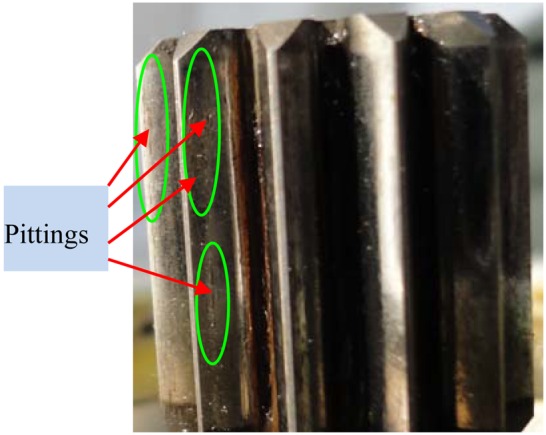
The defects in the sun gear of the planetary gearbox.

Figure 16(a,b,c) display the denoising results of customized multiwavelet using NC, GHM multiwavelet using INC and NC, respectively. As shown in Figure 16(a), periodic impulses can be accurately detected, while the weak impulse at 0.16 s are “over-killed” by the chosen threshold. Figure 16(b,c) display similar denoising results. Strong impulses are detected while weak fault features are “over-killed”. Moreover, GHM multiwavelet cannot accurately separate fault features and the noises are not eliminated completely. The planetary gearbox was disassembled. Figure 17 displays that four incipient pittings denoted as the arrows appear in three regions of 2 × 10 mm^2^. The field inspection verifies the fault diagnosis conclusion.

**Figure 18.**
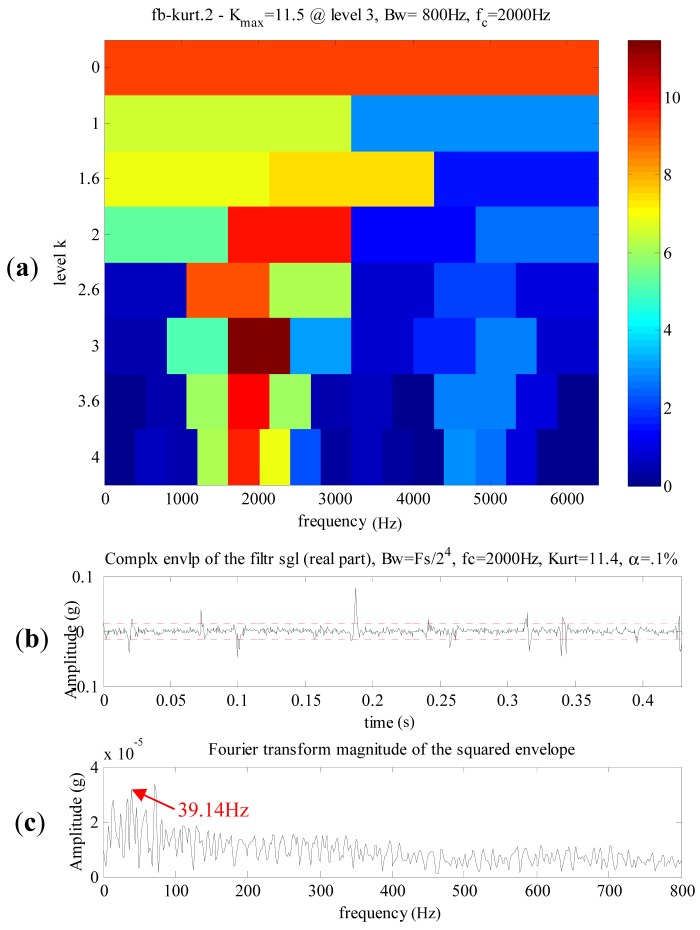
The analyzed results of the defective planetary gearbox signal using spectral kurtosis. (**a**) The Kurtogram. (**b**) The purified signal. (**c**) The envelop spectrum (low-frequency band).

The Kurtogram and the resulting signals of spectral kurtosis are illustrated in Figure 18, the maximum kurtosis is 11.5 at level 3, the central frequency is 2,000 Hz and the bandwidth is 800 Hz. The envelope of the filtering signal and its FFT spectrum are shown in Figure 18(b,c), respectively. Only strong impulses could be detected in the envelope of the filtered signal. Figure 18(c) displays a dominant frequency 39.14 Hz, which has a significantly difference with 43 Hz, the meshing frequency of the first stage in the planetary gearbox.

### 4.4. Results and Discussion

**Figure 19.**
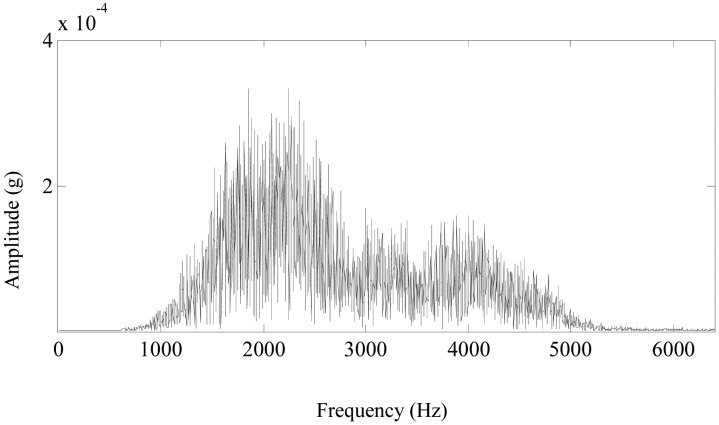
The Fourier spectrum of the denoising result using the proposed method.

1. From the planetary gearbox fault detections, it can be seen that multiwavelet denoising methods can effectively eliminate noise and improve the signal-to-noise ratio so as to outstand the fault features. Besides, the denoising methods using customized multiwavelets [shown in Figures 11(b) and 16(a) ] have a better result than the denoising methods using GHM multiwavelet. However, only the proposed method using the customized multiwavelets and the improved neighboring coefficients [shown in Figures 11(a) and 15] could effectively enhance the fault features. Thus, the appropriate multiwavelet functions for feature separation and the optimal threshold and length of neighbors for denoising are both important, neither of them could be dispensed with in planetary gearbox fault detections.
2. The computing times of these methods in planetary gearbox fault detections are listed in Table 3. All the tests of time consumption are carried out and recorded in Matlab software with the same computer platform of Intel Pentium Dual CPU E5200 @2.5 GHz and DDR2 memory 2 G. As it is known that every coin has two sides, the optimization of the customized multiwavelets has to take some time for the effective planetary gearbox fault detections. For example, a signal of length 4096 has to take about 2 minutes using the proposed method. In practice, the customized multiwavelets are suitable for a certain kind of fault. For example, the customized multiwavelets proposed in this paper are effective for detecting the pitting faults of other gears in planetary gearboxes, without constructing customized multiwavelets again. Therefore, the calculation efficiency of the proposed method is similar to other denoising methods using standard multiwavelets after the customized multiwavelets have been constructed.
3. As shown in Figure 14, the denoising result in time domain could accurately represent the shock impulses caused by the pittings of the sun gear. Its Fourier spectrum is shown in Figure 19, the frequency components mainly distribute at 1,000–3,000 Hz. The rotating frequency or the meshing frequency and their harmonics of the planetary gearbox cannot be detected in the spectrum. The spectrum is not so distinct as the denoising result in the time domain of Figure 14. Besides, the spectrums of denoising results using other methods are similar to the spectrum in Figure 19. Therefore, FFT is not effective to detect the pittings of the sun gear in the planetary gearbox.

## 5. Conclusions

As key components of the transmission train, planetary gearboxes play an important role in guaranteeing the normal operation of the satellite communication antenna. With their special gear transmission structure, planetary gearboxes exhibit complicated dynamic responses, which increase the difficulty of fault feature extractions for planetary gearboxes. It is proved that the customized multiwavelets which are similar to fault features and have excellent properties can achieve a good result in fault feature detections. The redundant symmetric lifting scheme is applied to produce customized multiwavelet functions. Moreover, the quotient of kurtosis and entropy is proposed to choose the optimal multiwavelets. On the basis of the local concentrated energy, the improved neighboring coefficients choose variant thresholds and sizes of neighbors at different decomposition levels. The proposed method incorporated customized multiwavelets and INC threshold. It was applied to the planetary gearbox fault detections. Experimental results showed that the proposed method could detect the pitting fault features on two neighboring teeth of the sun gear in a planetary gearbox.

## Figures and Tables

**Table 1.** The parameters of acceleration sensors.

**Model**	**Sensitivity (±10%)**	**Measurement Range**	**Frequency Range (±10%)**	**Non-Linerity**	**Sensing Element**
333B32	100 mv/g	±50,g pk	0.5–3,000 Hz	≤1%	Caremic

**Table 2.** The parameters of the planetary gearbox.

Type	The first stage	Sun gear tooth number	12
		Planet gear tooth number	36
		Ring gear tooth number	84
PLS142-32	The second stage	Sun gear tooth number	28
		Planet gear tooth number	28
		Ring gear tooth number	84

**Table 3.** The computing time of the methods in planetary gearbox fault detections.

**Method**	**Time in healthy planetary gearbox (s) (Signal length 5760)**	**Time in defective planetary gearbox (s) (Signal length 5760)**
Customized multiwavelets with INC	156.05 (*150.81*)	156.05 (*150.81*)
Customized multiwavelet with NC	155.18 (*150.81*)	155.18 (*150.81*)
GHM multiwavelet with INC	3.79	3.79
GHM multiwavelet with NC	3.71	3.71

The time in italics represents the cost for the optimization of the customized multiwavelets.

## References

[1] Yan R.Q., Gao R.X. (2008). Rotary machine health diagnosis based on empirical mode decompositon. J. Vib. Acoust..

[2] James M. (2002). Fourier series analysis of epicyclic gearbox vibration. J. Vib. Acoust..

[3] Vecchiato D. (2006). Tooth contact analysis of a misaligned isostatic planetary gear train. Mech. Mach. Theory.

[4] Lei Y.G., Kong D.T., Lin J., Zuo M.J. (2012). Fault detection of planetary gearboxes using new diagnostic parameters. Meas. Sci. Technol..

[5] Blunt D.M., Keller J.A. (2006). Detection of a fatigue crack ina UH-60A planet gear carrier using vibration analysis. Mech. Syst. Signal Process..

[6] Mosher M. Understanding Vibration Spectra of Planetary Gear Systems for Fault Detection.

[7] Hines J.A., Muench D.S., Keller J.A., Garga A.K. Effects of Time-Synchronous Averaging Implementations on HUMS Features for UH-60A Planetary Carrier Cracking.

[8] Inalpolat M., Kahraman A. (2009). A theoretical and experimental investigation of modulation sidebands of planetary gear sets. J. Sound Vib..

[9] Barszcz T., Randall R.B. (2009). Application of spectral kurtosis for detection of a tooth crack in the planetary gear of a wind turbine. Mech. Syst. Signal Process..

[10] Bartelmus W., Zimroz R. (2009). A new feature for monitoring the condition of gearboxes in non-stationary operating conditions. Mech. Syst. Signal Process..

[11] Bartelmus W., Zimroz R. (2009). Vibration condition monitoring of planetary gearbox under varying external load. Mech. Syst. Signal Process..

[12] Hameed Z., Hong Y.S., Cho Y.M., Ahn S.H., Song C.K. (2009). Condition monitoring and fault detection of wind turbines and related algorithms: A review. Renew. Sustain. Energy Rev..

[13] Lei Y.G., Lin J., He Z.J, Kong D.T. (2012). A method based on multi-sensor data fusion for fault detection of planetary gearboxes. Sensors.

[14] Daubechies I. (1992). Ten Lectures on Wavelets.

[15] Keinert F. (2004). Wavelets and Multiwavelets.

[16] Geronimo J.S., Hardin D.P., Massopust P.R. (1994). Fractal functions and wavelet expansions based on several scaling functions. J. Approx. Theory.

[17] Geronimo J.S., Hardin D.P., Massopust P.R. (1996). Construction of orthogonal wavelets using fractal interpolation functions. SIAM J. Math. Anal..

[18] Chui C.K., Lian J.A. (1996). A study of orthonormal multiwavelets. Appl. Numer. Math..

[19] Strela V., Strang G. (1995). Finite Element Multiwavelets. Approximation Theory, Wavelets and Applications.

[20] Khadem S.E., Rezaee M. (2003). Development of vibration signature analysis using multiwavelet systems. J. Sound Vib..

[21] Yuan J., He Z.J., Zi Y.Y., Liu H. (2009). Gearbox fault diagnosis of rolling mills using multiwavelet sliding window neighbouring coefficient denoising and optimal blind deconvolution. Sci. China Ser. E Technol. Sci..

[22] Li Z., He Z.J., Zi Y.Y., Jiang H.K. (2008). Rotating machinery fault diagnosis using signal-adapted lifting scheme. Mech. Syst. Signal Process..

[23] Sweldens W. (1996). The lifting scheme: A custom-design construction of biorthogonal wavelets. Appl. Comput. Harmonic Anal..

[24] Sweldens W. (1998). The lifting scheme: A construction of second generation wavelet. SIAM J. Math. Anal..

[25] Wang X.D., Zi Y.Y., He Z.J. (2009). Multiwavelet construction via an adaptive symmetric lifting scheme and its applications for rotating machinery fault diagnosis. Meas. Sci. Technol..

[26] Yuan J., He Z.J., Zi Y.Y. (2010). Gear fault detection using customized multiwavelet lifting schemes. Mech. Syst. Signal Process..

[27] Mallat S. (2003). A Wavelet Tour of Signal Processing.

[28] Wang X.D., Zi Y.Y., He Z.J. (2011). Multiwavelet denoising with improved neighboring coefficients for application on rolling bearing fault diagnosis. Mech. Syst. Signal Process..

[29] Xia X.G., Geronimo J.S., Hardin D.P., Suter B.W. (1996). Design of prefilters for discrete multiwavelet transform. IEEE Trans. Signal Process..

[30] Strela V., Heller P.N., Strang G., Topiwala P., Heil C. (1996). The application of multiwavelet filter banks to signal and image processing. IEEE Trans. Image Process..

[31] Bradley A.P. Shift-Invariance in the Discrete Wavelet Transform.

[32] Bulat J. Image Compression Using Lifted Wavelet Packets.

[33] Cai T.T., Silverman B.W. (2001). Incorporating information on neighboring coefficients into wavelet estimation. Sankhya.

[34] Chen G.Y., Bui T.D. (2003). Multiwavelets denoising using neighboring coefficients. IEEE Signal Process. Lett..

[35] Lin J., Qu L.S. (2000). Feature extraction based on Morlet wavelet and its application for mechanical fault diagnosis. J. Sound Vib..

[36] Antoni J. (2006). The spectral kurtosis: A useful tool for characterising non-stationary signals. Mech. Syst. Signal Process..

[37] Antoni J. (2007). Fast computation of the kurtogram for the detection of transient faults. Mech. Syst. Signal Process..

